# *Ofeleein i mi Vlaptin*—Volume II: Immunity Following Infection or mRNA Vaccination, Drug Therapies and Non-Pharmacological Management at Post-Two Years SARS-CoV-2 Pandemic

**DOI:** 10.3390/medicina58020309

**Published:** 2022-02-17

**Authors:** Jannis Kountouras, Dimitra Gialamprinou, Georgios Kotronis, Apostolis Papaefthymiou, Eleftheria Economidou, Elpidoforos S. Soteriades, Elisabeth Vardaka, Dimitrios Chatzopoulos, Maria Tzitiridou-Chatzopoulou, Dimitrios David Papazoglou, Michael Doulberis

**Affiliations:** 1 Second Medical Clinic, School of Medicine, Ippokration Hospital, Aristotle University of Thessaloniki, 54652 Thessaloniki, Central Macedonia, Greece; appapaef@hotmail.com (A.P.); evardaka@aqua.teithe.gr (E.V.); chatzopoulosdimitrios@gmail.com (D.C.); mariatzitiridou@gmail.com (M.T.-C.); doulberis@gmail.com (M.D.); 2 Second Neonatal Department and NICU, Papageorgiou General Hospital, Aristotle University of Thessaloniki, 56403 Thessaloniki, Central Macedonia, Greece; gialamprinou@gmail.com; 3 Department of Internal Medicine, General Hospital Aghios Pavlos of Thessaloniki, 55134 Thessaloniki, Central Macedonia, Greece; georg.kotronis@gmail.com; 4 Department of Gastroenterology, University Hospital of Larisa, Mezourlo, 41110 Larisa, Thessaly, Greece; 5 School of Economics and Management, Healthcare Management Program, Open University of Cyprus, Nicosia 12794, Cyprus; eleftheria.economidou@gmail.com (E.E.); elpidoforos.soteriades@ouc.ac.cy (E.S.S.); 6 Department of Environmental Health, Harvard T.H. Chan School of Public Health, Environmental and Occupational Medicine and Epidemiology (EOME), Boston, MA 02115, USA; 7 Department of Nutritional Sciences and Dietetics, School of Health Sciences, International Hellenic University, 57400 Thessaloniki, Central Macedonia, Greece; 8 Midwifery Department, School of Healthcare Sciences, University of West Macedonia, Koila, 50100 Kozani, Central Macedonia, Greece; 9 Department of Cardiovascular Surgery, Inselspital, Bern University Hospital, University of Bern, 3010 Bern, Switzerland; papazoglou2019@gmail.com; 10 Division of Gastroenterology and Hepatology, Medical University Department, Kantonsspital Aarau, 5001 Aarau, Switzerland

**Keywords:** COVID-19, SARS, Hippocrates, *ofelein i mi vlaptin*, primum non nocere, vaccination, mRNA, drug therapy

## Abstract

The persistence of the coronavirus disease 2019 (COVID-19) pandemic has triggered research into limiting transmission, morbidity and mortality, thus warranting a comprehensive approach to guide balanced healthcare policies with respect to people’s physical and mental health. The mainstay priority during COVID-19 is to achieve widespread immunity, which could be established through natural contact or vaccination. Deep knowledge of the immune response combined with recent specific data indicates the potential inferiority of induced immunity against infection. Moreover, the prevention of transmission has been founded on general non-pharmacological measures of protection, albeit debate exists considering their efficacy and, among other issues, their socio-psychological burden. The second line of defense is engaged after infection and is supported by a plethora of studied agents, such as antibiotics, steroids and non-steroid anti-inflammatory drugs, antiviral medications and other biological agents that have been proposed, though variability in terms of benefits and adverse events has not allowed distinct solutions, albeit certain treatments might have a role in prevention and/or treatment of the disease. This narrative review summarizes the existing literature on the advantages and weaknesses of current COVID-19 management measures, thus underlining the necessity of acting based on the classical principle of “*ofeleein i mi vlaptin*”, that is, to help or not to harm.

## 1. Introduction

Coronavirus disease 2019 (COVID-19) is caused by the novel coronavirus SARS-CoV-2. It was first reported in Wuhan, China, in December 2019, and since then it has emerged as a global pandemic [1]. According to officially published statistical data of the World Health Organization (WHO), on 31 December 2021 a total of 281,808,270 positive cases and 5,411,759 deaths have been globally recorded [2]. Acknowledged preconditions that contribute significantly to COVID-19 “expansion” include the high potential of “human-to-human” virus transmission as well as the existence of an immunologically naïve population [3].

In the battle against this universal threat, a plethora of preventive as well as therapeutic measures have been implemented with varying degrees of efficacy, effectiveness and safety. The main representatives comprise the available vaccines, pharmacological agents and the non-pharmacological interventions [4]. In our previous review [1], we introduced the classical principle of Hippocrates (Ιπποκράτης, 460–370 BC) “*ofelein i mi vlaptin*” (*ὠφελέειν ἢ μὴ βλάπτειν*), that is, either to help or not to harm the patient. This fundamental ethical approach guides the professional practice of all physicians and is also applicable in the management of COVID-19, among other contexts.

It is common knowledge that severity as well as mortality risk of COVID-19 directly correlates with age [5], with mortality risk being negligible for young patients, whereas it increases significantly for elderly, especially if they are 65 years or older [6]. Furthermore, comorbidities constitute an additional acknowledged component of COVID-19 severity and mortality [7,8]. Moreover, additional predictors of mortality at admission to an intensive care unit (ICU) have been identified as acute respiratory distress syndrome (ARDS), interleukin (IL)-6, serum albumin, and D-dimer levels, as well as chest CT severity score [9]. The aforementioned parameters have been further utilized within the developed AIDA score and validated in an additional sample of 304 patients admitted to ICU due to the severe form of COVID-19 [10].

Given this stratification of risk for COVID-19 patients, a potential favorable outcome should always be weighed against the possible adverse effects of each preventive and therapeutic strategy. Thus, within this narrative literature review, we discuss, based on current available scientific evidence and through the prism of “*ofelein i mi vlaptin*”, the hot topics of acquired immunity following natural infection or mRNA vaccination. In addition, we present the state of the art in the main current pharmacological treatments and also briefly cover non-pharmacological management.

## 2. Immunity after SARS-CoV-2 Infection versus Post Vaccination

Exposure to both whole virus in terms of a natural SARS-CoV-2 infection and to spike proteins through mRNA vaccination leads to acquired immunity, at least to a certain extent.

A plethora of studies stemming from different populations and countries supports, with emerging evidence, the robust and long-term immunity of previously infected individuals. For instance, an epidemiological study was recently conducted in Sweden [11] and included a sample of 1884 healthcare workers and 51 hospitalized COVID-19 patients. The authors reported that all COVID-19 patients and the vast majority of healthcare workers (96%) who were anti-spike IgG-positive at the beginning of the study, maintained their positivity eight months later. Moreover, circulating SARS-CoV-2 specific memory T lymphocyte responses were recorded in 88% of COVID-19 patients and 63% of seropositive healthcare workers, respectively. Furthermore, a cumulative incidence of 1% SARS-CoV-2 infection, confirmed by means of PCR, was demonstrated among anti-spike IgG-positive health-care workers compared with 23% among anti-spike IgG-negative healthcare workers, yielding a satisfactory protective effect of 95.2% [11]. Furthermore, a French study group, by including a prospectively followed sample of 1309 individuals, displayed that long-term titers of the so-called “specific receptor binding domain” (RBD) IgG persist up to 13 months post SARS-CoV-2 infection. This phenomenon may decrease according to the authors, leading to a potential of reinfection in convalescent COVID-19 patients by variants D614G and B.1.1.7 [12].

Of note, RBD is known to be particularly immunogenic and triggers SARS-CoV-2-neutralizing antibody production. On the other hand, antibodies against the N-terminal domain (NTD) of the spike protein exert an enhancement of binding of the virus to the angiotensin-converting enzyme (ACE-2) receptor and therefore reinforce SARS-CoV-2 infectivity [13]. This phenomenon was demonstrated for the original Wuhan strain D614G only in vitro. Yahi et al. [13] observed that enhancing NTD antibodies appeared to have a higher affinity for the currently widespread Delta variants compared to the Wuhan strain. In detail, the authors demonstrated that enhancing antibodies favors the binding of the spike trimer to the host cell membrane by clamping the NTD to lipid raft microdomains. This stabilizing mechanism might promote conformational modification, which triggers the demasking of the RBD. Since NTD is also targeted by neutralizing antibodies, the authors proposed that the balance between neutralizing and facilitating antibodies in vaccinated individuals is in favor of a neutralization process only for the original Wuhan strain (D614G). Nevertheless, as far as Delta variants are concerned, neutralizing antibodies appear to have a lesser affinity for the spike protein, whereas facilitating antibodies display a strikingly increased affinity. Thus, antibody-dependent enhancement (ADE) may be a concern for people receiving vaccines based on the original Wuhan strain spike sequence, such as the current mRNA vaccines [13]. A variety of mechanisms has been postulated to be involved in ADE, which appears to be a limiting factor in the development of new vaccines and therapeutic approaches [14,15]. The rationale is to drive both high-specificity humoral and cellular immunity while avoiding undesirable consequences, such as ADE.

Beyond the existence of protective antibody titers, longed-lived bone marrow plasma cells are believed to provide a further necessary component of immunity following SARS-CoV-2 infection. In particular, *Nature* published a relevant study, which demonstrated that convalescents after COVID-19 with oligosymptomatic course had detectable titers of anti-spike antibodies for at least 11 months. Additionally, the aforementioned antibodies correlated with the frequency of spike protein-specific plasma cells from isolated bone marrow aspirates of COVID-19 convalescents 7–8 months before, a phenomenon not observed in healthy volunteers. It was also revealed that this binding attribute of plasma cells to spike proteins is quiescent as part of a stable compartment [16].

Interestingly, the IgG antibodies against spike proteins of all patients up to seven months after COVID-19 show in vitro significant neutralizing activities. Additionally, interferon γ-producing CD4+, as well as CD8+ cells, were upregulated upon SARS-CoV-2 antigen stimulation [17]. Further encouraging results regarding long-term neutralizing antibodies have been reported by an American study; 90% of SARS-CoV-2-positive individuals triggered both spike and nucleocapsid immunological responses, and all but one of these had persistent antibody titers at six-month follow-up. Pseudoviral neutralization activity was widespread among participants, did not lessen with the elapse of time and correlated with clinical antibody assays [18].

Considering the reinfection of convalescent individuals, an Austrian retrospective study (*n* = 14,840) reported a relatively low reinfection rate (0.27%) among individuals with COVID-19 and deduced that protection against SARS-CoV-2 following a natural infection is comparable to the highest available estimates of vaccine efficacy [19]. Comparable results were also yielded by another retrospective study [20] from California with a sample size of *n* = 75,149, where the overall incidence of reinfection after 270 days was as low as 0.8%. Likewise, a prospective Italian study (*n* = 546) investigated reinfection rates of SARS-CoV-2 up to ten months post infection. The authors concluded that the reinfection rate was as high as 1.1%, with a median of 9 months after first infection and its course mild or asymptomatic [21]. An even lower reinfection rate was reported by Utah investigators. In the relevant retrospective cohort, with a sample size of 23,176 COVID-19 patients, the rate of reinfection was as low as 0.04% [22]. In addition, an Iranian study reported a 0.33% re-infection/reactivation rate at one year post SARS-CoV-2 infection [23].

To sum up, beyond the isolation of anti-SARS-CoV-2 antibodies up to 13 months after infection, the organism is equipped with memory B cells which differentiate rapidly to specialized plasma cells for the production of antibodies, as well as specific T lymphocytes, which exert cytotoxic activity against the virally infected cells and support a low reinfection rate [24].

On the other hand, mRNA vaccines may induce short-term two to four-fold higher titers of neutralizing antibodies compared to natural infection [25]. Obtained immunity after BNT162b2 mRNA vaccination might be a matter of concern, regarding the longevity of producing antibodies. In this context, a recent study demonstrated that antibody titers of vaccinated individuals fall rapidly month by month compared to those of convalescent patients. In particularly, Israel et al. [26] compared 2653 fully vaccinated participants (BNT162b2) with 4361 previously infected patients in terms of decreasing antibodies with the elapse of time. They reported a decrease of antibody titers up to 40% for each subsequent month (BNT162b2 arm), whereas the recovered participants’ titers dropped by less than 5% per month. Six months after vaccination with BNT162b2, a total of 16.1% subjects had antibody levels below the seropositivity threshold of <50 AU/mL compared with only 10.8% of SARS-CoV-2-recovered participants who fell below the <50 AU/mL threshold 9 months after infection [26].

Moreover, recent evidence indicates that, compared to vaccination with mRNA, natural SARS-CoV-2 infection triggers a more robust humoral immune response in unexposed individuals. Additionally, the same study group deduced that functional neutralizing antibody tests are more relevant indicators than the mere presence or absence of relevant binding antibodies [27]. Furthermore, a recent study of hospital workers with a sample size of 273 individuals reported that out of 86% of individuals with antibody levels higher than 4160 AU/mL six weeks after the second shot only 40% retained their titers twelve weeks post vaccination [17].

Similar results were reported by a Japanese prospective study that recruited 225 healthy subjects to be vaccinated with two doses of BNT162b2. The aim of the study was the estimate the half-life of neutralizing activity and the time needed for the loss of detectable neutralizing activity (both expressed as averages). The investigators reported that BNT162b2 efficacy was likely to be fall below the detection limit six to seven months after the first vaccination dose. Secondly, a few moderate-level neutralizing antibody-containing serums failed to prevent infection with SARS-CoV-2 [28].

Another recent study, by estimating the antibody and memory T lymphocyte responses following a two-dose BNT162b2 vaccine in 122 volunteers for up to 6 months showed that the mRNA vaccine provoked a robust antibody response to SARS-CoV-2 and five VOCs at 1 week post vaccination, which subsequently declined. Spike-specific memory T lymphocyte responses, while evident in the majority (87%), were lesser in participants with higher T lymphocyte immunosenescence, thereby signifying the worsening of the vaccine response and therefore possibly necessitating booster vaccination [29]. Conversely, for instance, vaccination against hepatitis B virus, the structural protein of which appears to contain an RNA-polymerase capable of forming the adult coronavirus [30], induces long-term immunity and cell-mediated immune memory for up to two decades without requiring booster vaccination [31]. Memory T lymphocyte responses display a form of prominent immunodominance for chronic and self-limited viral infections and this form may persist over several decades in the absence of antigen stimulation [32].

Additionally, older Canadian adults appear to exhibit reduced magnitude and durability of humoral immune responses after mRNA vaccination [33]. Synoptically, the authors of this study reported that after the elapse of three months following the second mRNA vaccination, all humoral responses were significantly reduced in all individuals and remained significantly lower among aged adults, who also exhibited reduced ACE2 competition activity and binding antibodies against the Delta variant.

Worth mentioning, also, are the reported poor titers of anti-spike IgG antibodies in several patient populations. For instance, liver transplant patients or those with chronic liver disease are known to perform poorly in terms of antibody production following vaccination. A similar phenomenon has been observed for kidney transplant recipients who were treated with belatacept; they exhibited only a weak antibody response to three doses of mRNA vaccine [34]. The same result was obtained in a Greek prospective cohort study in patients with hematological malignancies [35]. Inselspital of Berne in Switzerland showed in a clinical trial which recruited patients taking medications targeting CD20 (i.e., rituximab or ocrelizumab) that such patients are characterized by impaired cellular and humoral immunity when vaccinated with Moderna mRNA-1273 or Pfizer–BioNTech BNT162b2 [36].

Interestingly, very recent data indicate that, in vitro, the SARS-CoV-2 spike protein considerably inhibits the DNA damage repair pathway, which is necessary for efficient V(D)J recombination in adaptive immunity, thereby impairing adaptive immune response and implying a potential side effect of full-length spike-based vaccines [37]; spike proteins inhibit DNA damage repair by inhibiting the recruitment of the main DNA repair proteins BRCA1 and 53BP1 to the damage site [37]. On the other hand, mutations of these repair genes seem to drive oncogenesis [38]. In this respect, certain identified genes, including E2F transcription factors and *retinoblastoma tumor-suppressor* gene (*RB1*), modulated by COVID-19 infection, appear to be involved in SARS-CoV-2-related carcinogenesis [39]; genetic variants of IL12RB1 confer genetic susceptibility to SARS-CoV-2 infection [40]; and mutations to RB1 and/or components regulating the cyclin dependent kinase-RB-E2F signaling pathway have been recognized in almost every human cancer [41]. In this setting, mutations in genes (*hMSHS2*, *hMLS1*, *hPMS1*, *hPMS2*) that also control the DNA mismatch repair process have been involved in oncogenesis. If “mismatch repair” (MMR) genes are mutated, respective errors may affect genes important to cancer progression, such as tumor-suppressor genes, leading to the development of malignancies, such as upper and lower gastrointestinal tract cancers [42]. In this regard, recent evidence suggest that administration of a SARS-CoV-2 vaccine (BNT162b2 mRNA vaccine) can induce angioimmunoblastic T cell lymphoma, a sub-type of peripheral T cell lymphoma [43] progression [44]. It is important to note that such lymphomas exhibit TP53 mutations in association with p53 (87%) expression [45,46]. Common mutations of such lymphomas are those related to the DNA repair–TP53 pathway (64%) and TP53 mutation, which is significantly associated with symptomatology and is a risk factor for patient survival [47]. Moreover, the gene *TP53*, which encodes the tumor-suppressor protein p53, is mutated in about 50% of malignancies, and p53 acts as a transcription factor to activate subsets of target genes which carry out cell fates, including DNA repair [48]. DNA damage-repair gene mutations are enriched in TP53 tumors, and *TP53* or DNA damage-repair gene mutations are frequently detected in malignancies with high-risk features [49]. However, the aforementioned concerns require further investigation, the true potential of such a hypothesized oncogenetic cascade of full spike protein being in need of elucidation.

A further concern regarding post-vaccination immunity is the reported liver autoimmume disorder development. In particular, new evidence indicates a possible association between autoimmune hepatitis (AIH) and COVID-19 vaccination [50]. Adverse events of the vaccine are possible, and liver disorders following vaccination represent an interesting clinical problem. Some investigators propose that an abnormal immune response is the ultimate cause of AIH [51], although it is not possible to exclude the existence of pre-vaccination autoimmunity. After vaccination, there is also the probability of excessive blood viscosity, which might also be a trigger factor for liver disorders [51].

Recent data also indicate that poor antibody responses for post-mRNA vaccines are observed in 61% of liver transplant recipients and 24% of those with chronic liver disease [52] and that immune responses to SARS-CoV-2 mRNA vaccination may also be impaired in patients with malignant disease [53].

Lastly, regarding the latest known variant, “Omicron”, preliminary data claim that the Pfizer–BioNTech vaccine’s efficacy might be significantly lower against this variant, with a 41-fold lower titer of neutralizing antibodies compared with the initial variants at the beginning of the pandemic (with spike protein substitution D614G) [54]. Moreover, in an official announcement, Pfizer admitted that with the Omicron variant “more than a 25-fold reduction in neutralization titers” was anticipated and that two vaccines “may not be sufficient to protect against infection with the Omicron variant” [54].

All in all, regarding the mRNA vaccinations, it has to be stated that, despite the several aforementioned concerns that have been raised, they still seem to be possibly effective, particularly for high-risk segments of the aged with/or comorbidities population [55,56,57,58].

## 3. COVID-19 Evidence-Based Treatments

Beyond the outstanding research aiming at the discovery of potential safe and effective vaccines against COVID-19, a further global endeavor points to developing new pharmacological regimes or utilizing existing ones which, ideally, could provide effective therapeutic approaches for COVID-19 patients with clinical symptoms. Given the fact that the development of new drugs may take decades, an impressive plethora of diverse agents have been evaluated as potential treatments with varying degrees of efficiency and effectiveness.

Specifically, there is a long list of pharmaceutical agents that have been studied for the management of COVID-19, either in outpatient settings alone or in both in- and out-of-hospital treatment settings. Among others, worth mentioning are well-established older drugs which have been administered previously for other indications, such us colchicine, azithromycin, doxycycline, lopinavir/ritonavir, proxalutamide and ivermectin [59,60].

For instance, ivermectin is a well-known antiparasitic drug with a very attractive profile, since it combines adequate safety and particular cost-effective outcomes. It is considered that around one third of the world’s population, particularly patients stemming from low- and middle-income countries, have been treated with ivermectin for worm, scabies and lice infections [59,60]. There is emerging evidence that ivermectin may also exhibit anti-inflammatory and antiviral properties. It has been demonstrated previously to have antiviral activity against Zika, Dengue and Yellow Fever [61].

An early in vitro study by Caly et al. reported a specific antiviral effect of ivermectin against SARS-CoV-2, an action which was interpreted as a possible blockage of the nuclear import of viral proteins suppressing normal immune responses [62]. Further encouraging results were demonstrated by a French in vivo study in which ivermectin administration attenuated both clinical and immunological outcomes of hamsters infected with SARS-CoV-2 [63]. The authors performed transcriptomic analyses of pulmonary tissue to interpret their results. Synoptically, they attributed the favorable outcome to a profound lessening of type I interferon responses with a parallel decrease of pro-inflammatory IL-6/IL-10 ratios in tissues as well as promotion of M2 macrophage polarization [63]. Furthermore, more than 20 randomized controlled trials (RCT) [60] and eight meta-analyses [61,64,65,66,67,68,69] have been conducted, with an overall reported conclusion being a major reduction of COVID-19 morbidity and mortality; such RCTs reported significant decreases in mortality accompanied by significant decreases in the length of viral clearance and clinical recovery. Several controlled prophylaxis trials showed a lower risk of contracting SARS-CoV-2 to be associated with regular usage of ivermectin [70]. Therefore, in view of the worldwide emergency of COVID-19, with mutant viral strains and/or vaccination denials, ivermectin may serve as an effective preventive and therapeutic strategy against the current pandemic if introduced for both prevention and treatment of COVID-19 [60,70]. Nevertheless, further relative studies are needed since low to moderate certainty of evidence has to be acknowledged for the available meta-analyses [61]. At least two recent meta-analyses reported a negative result [71,72], which might be attributed to differences in methodology and the populations included. In any case, it should be clearly stated that, like any other medication, judicious use of ivermectin could be considered for selected COVID-19 patients, since inappropriate or excessive prescription of this agent may potentially lead to undesirable effects, including drug interactions [73,74]. In this respect, it should be acknowledged that rare cases of ivermectin toxicity have been recently reported [75]. The latter might be attributed to homozygosity for a nonsense mutation in gene *ABCB1* [76]. For instance, in *ABCB1*-knockout mice, ivermectin provokes neurological defects which may be fatal [76], analogous to the known toxicity in certain canine breeds, such as collies [76].

Proxalutamide is another promising pharmacological agent in the battle against COVID-19. It is a potent non-steroidal androgen receptor antagonist (second-generation) [77]. Of note, androgen signaling pathways seem to enact a key role in terms of SARS-CoV-2 infectivity. In particular, viral entry to host cells is mediated through the priming of SARS-CoV-2 spike proteins by the androgen-promoted enzyme transmembrane serine protease 2 (TMPRSS2). Structural modifications of spike proteins ameliorate binding of SARS-CoV-2 to ACE-2 and assist intracellular viral entry. It is therefore reasonable to conclude that targeting the transcription of TMPRSS2 with antiandrogens can affect viral cell entry [77]. As with ivermectin, results from RCTs [77,78], as well as a network of meta-analyses, support the favorable effect of proxalutamide on COVID-19 in terms of severity and mortality, accompanied by augmented viral clearance [59] and lower hospitalization rates [77].

Colchicine is an old, safe, inexpensive but strong anti-inflammatory medication with favorable pleiotropic and cardioprotective effects targeting the excessive immune system activation following SARS-CoV-2 infection. These effects result mainly via inhibition of neutrophil activity, as well as IL-1β, thereby preventing the cytokine storm [79,80,81,82]. Moreover, colchicine administered at standard doses has been shown to attenuate the connection between inflammation and thrombosis, therefore reducing the thrombogenic risk in COVID-19 patients, without affecting the otherwise physiological process of thrombosis [79]. More specifically, during the COVID-19 pandemic, colchicine has been considered a good therapeutic option owing to its effects on the parts of the immune system involved in COVID-19 infection and acute respiratory distress syndrome, including its effects on the chemotaxis of inflammatory cells, such as neutrophils and monocytes, and the intracellular transportation of vesicles. Colchicine also inhibits the inflammasome, expression of different molecules involved in leukocytes binding to endothelial cells and the recruitment of mononuclear cells and neutrophils to inflamed tissue [83]. It acts as a pyrin domain-containing protein 3 (NLRP3) inflammasome inhibitor and additionally diminishes IL-1β synthesis, thereby delaying and reducing the extent of immune system activation that could drive the development of ARDS/ALI in COVID-19 patients [84,85]. Specifically, it prevents microtubule assembly and results in subsequent disruption of multiple inflammatory pathways, such as the aforementioned NLRP3 inflammasome activation, microtubule-based inflammatory cell chemotaxis, generation of leukotrienes and cytokines and phagocytosis. Moreover, colchicine could inhibit the viroporin E-mediated activation of the inflammasome during COVID-19 infection, with subsequent impairment of the production of IL-1β, which results in the abolishment of the secretion of IL-6 and tumor necrosis factor (TNF)-α, leading to a reduced recruitment of neutrophils and macrophages [79]. It also reduces the production of reactive oxygen species and α-defensin [79]. Furthermore, colchicine inhibits neutrophil–platelet aggregation and increases the levels of protein C [86], highlighting its anti-thrombotic properties [79]. Such favorable anti-thrombotic attributes of colchicine are of exceptional interest, particularly when an increasing body of evidence reports post-vaccination cases of serious thromboembolic complications, such as vaccine-associated thrombocytopenia and thrombosis [87,88].

The safety profile of colchicine at a dose of 0.5–2 mg per day has been demonstrated by decades of observational studies and clinical practice [89]. Therefore, due to its beneficial effects, safety profile and availability, colchicine appears to be an effective drug for COVID-19 treatment. In this regard, a recent study focusing on the role of colchicine in preventing severe COVID-19 complications revealed a potential favorable effect in delaying clinical deterioration in admitted patients [85]. Moreover, a meta-analysis of six small studies showed a significantly favorable impact of colchicine in reducing mortality among COVID-19 patients [90]. Likewise, an updated meta-analysis of eight studies (a total of 5259 COVID-19 patients) showed that colchicine reduced mortality, and patients who received colchicine tended to have a lower risk of requiring mechanical ventilation [91]. Another recent meta-analysis which included 17,205 COVID-19 patients also showed significant reduced mortality in patients who received colchicine therapy [92]. Discontinuation of trials owing to colchicine-related side effects was rare. Further consideration of colchicine for prevention against COVID-19, as well as for treatment of COVID-19 complications, and assessment of meta-analyses signifying a mortality risk reduction in participants treated with colchicine, currently available data have shown colchicine’s potential in treating SARS-CoV-2-infected patients [93]. Nevertheless, it ought to be noted that high doses of colchicine have been previously linked to increased toxicity [94].

Concerning the safety profile of colchicine, a recent pooled meta-analysis of patients with COVID-19 reported that colchicine is a well-tolerated drug and has a good safety profile except for a higher chance of diarrhea [95]. Similar findings were observed in another meta-analysis carried out pre-COVID-19 [96].

Chloroquine (CQ), along with its derivative hydroxychloroquine (HCQ) with an extra hydroxyl group, has a long history (~50 years) as an effective antimalarial drug with immunomodulatory, anti-thrombotic, anti-metabolic, autophagic, apoptotic and anti-neoplastic properties [97,98]. In the recent past, the effectiveness of these drugs has been studied in relation to a diversity of acute infections, including Zika, influenza A H5N1, Ebola, dengue, and chikungunya, as well as chronic viral infections, including hepatitis C and human immunodeficiency viruses [99,100,101]. CQ and HCQ have been suggested for the prevention and treatment of COVID-19 due to their in vitro antiviral activity against the severe acute respiratory syndrome associated with coronavirus 2 (SARS-CoV-2) [102,103,104] and their potential for suppressing the release of pro-inflammatory cytokines [105]. Both drugs have been reported to block multiple steps in the SARS-CoV-2 life cycle, for instance, viral binding and entry into cell, release of the viral genome, viral replication, virion assembly and budding [102]. CQ and HCQ are relatively safe and the most frequent side effects include dermatological alterations, pruritus and gastrointestinal symptoms, occurring in up to 10% of patients. The most severe side effects include cardiotoxicity, neuromyopathy of proximal muscles and irreversible retinopathy, which have very low incidence [106]. HCQ displays a superior safety profile as compared with CQ [98,102]. Therefore, HCQ or CQ provide a promising approach for the treatment of COVID-19. In this respect, recent randomized controlled trials suggest potential benefits in clinical outcomes, thereby signifying that CQ and HCQ are promising therapeutic agents against COVID-19 pneumonia [107]. A meta-analysis of clinical reports showed improvement in clinical and virological outcomes for patients using CQ [108]. Moreover, an evidential synthesis based on observational studies showed a 7–33% reduced mortality in hospitalized patients with COVID-19 using a lower dosage of HCQ [109]. Likewise, in a meta-analysis of 11 RCTs, the use of HCQ was not linked with increased mortality. It was also associated with a 20% reduced risk of mortality in a meta-analysis of 25 observational studies [110]. Nevertheless, Mehra et al. conducted a disputed multinational registry analysis that revealed an increased risk of death with HCQ in COVID-19 patients. Interestingly, this research article was retracted soon after publication [111]. This discrepancy between RCTs and observational studies could mainly be attributed to the lower median doses used, which seemed to provide beneficial effects in terms of mortality risk in COVID-19 patients.

It is important to note that many clinicians have reported positive outcomes in treating COVID-19 patients using these drugs owing to their low cost, long history of use and availability. It is expected that studies now underway will provide more definitive answers. A recent large-scale ambulatory series study examining HCQ plus azithromycin as a combined therapy established the efficacy of early COVID-19 outpatient treatment without HCQ-related serious cardiac side effects; early ambulatory treatment of COVID-19 with this combined regimen was associated with very low mortality and improved survival when compared with other regimens [112]. Azithromycin, an antibacterial of the macrolide, seems to exhibit potential antiviral and immunomodulatory therapeutic efficacy, in monotherapy and in combination, for the treatment of SARS-CoV-2 infection [113]. However, since the described side effects of these agents when introduced in combination with azithromycin in patients with comorbidities have raised significant safety concerns, additional high-quality randomized clinical trials are needed to clarify such concerns [107]. Of note, trials also investigated these drugs in combination with zinc supplementation [114].

Other antibiotics more frequently prescribed during the initial peak of COVID-19 in outpatient settings include doxycycline, cefpodoxime and sulfamethoxazole–trimethoprim [115].

Glucocorticoids, mainly dexamethasone—a widely available and inexpensive medication that could be made available to COVID-19 patients globally—have been widely used as immunomodulators aiming to reduce lung parenchymal inflammation and subsequent lung tissue damage that may lead to respiratory failure. In this regard, the utility of glucocorticoids in improving outcomes has been put under scrutiny, with different clinical views expressed during the first months of the pandemic [116,117]. A small, open-label randomized trial provided weak evidence that a 6-day course of intravenous methylprednisolone offered a benefit in severe COVID-19 cases in terms of significantly reducing the risk of death, admission to an intensive care unit (ICU) or the need for non-invasive ventilation as compared with the standard-of-care group [118]. A meta-analysis of seven RCTs also showed a beneficial effect of systemic corticosteroids in lessening the 28-day mortality in individuals with severe COVID-19 [119].

Specifically, administration of dexamethasone has been reported to correspond to dose-dependent inhibition of pro-inflammatory cytokine production, such as IL-12, but not to affect anti-inflammatory cytokines, such as IL-10 [120]. Thus, it might be beneficial for treating the SARS-CoV-2-induced cytokine storm [121]. In this regard, a large randomized controlled trial suggests that administration of dexamethasone to hospitalized patients who are unable to breathe independently may significantly improve survival outcomes [122] given the results obtained for the dexamethasone arm as compared to standard of care alone. The above glucocorticoid, administered for a 10-day course, was found to provide favorable effects by means of decreasing the 28-day mortality risk in patients requiring respiratory support. However, there was no benefit for patients not requiring respiratory support and, in contrast, a potential harm was noted in this subgroup [122]. Likewise, a meta-analysis indicates that dexamethasone may significantly improve outcomes among COVID-19 hospitalized patients associated with severe respiratory complications [123]. Data from a recovery trial also indicates that dexamethasone significantly reduces SARS-CoV-2-related deaths (by around 30% among patients receiving mechanical ventilation and by around 20% among those receiving oxygen alone) [124]. Given the aforementioned available evidence, dexamethasone currently appears to be the most promising treatment for severe COVID-19.

The prospect of managing SARS-CoV-2 infection by means of cytokine inhibition has been widely discussed and IL-1 together with IL-6 and TNF-α are considered to play key roles in the cascade of cytokine release during generalized immune system activation. An extensive literature has been published regarding the potential beneficial effects of IL-1 inhibition in eliminating the aforementioned ‘’cytokine storm’’. Cytokine storm syndrome, also referred to as cytokine release syndrome (CRS), is the process by which an increase in inflammation precipitates the excessive activation of the coagulation pathways and disrupts the integrity of vascular permeability, causing endothelial injury [125,126]. CRS-related dysregulation of immune responses may induce epithelial and endothelial cell apoptosis and vascular leakage, T lymphocyte suboptimal responses, dysfunctional and overactive macrophages infected with SARS-CoV-2, as well as dysfunctional tissue homeostasis, all of which appear to contribute to the pathophysiology and severity of macrophage activation storms (MASs), resulting in acute respiratory distress syndrome (ARDS) [125,127]. MAS is indirectly stimulated by IL-1 [128], so that inhibiting IL-1, and thereby MAS and ARDS, appears to be a relatively significant preventive target point. Additionally, augmented IL-1 release in viral infections leads to lung and tissue inflammation, fever and fibrosis, with consequent respiratory complications in the COVID-19-infected host [129]. Regarding the pro-inflammatory pathways involved in the development of fibrosis, it seems that SARS-CoV-2 employs different molecular pathways to activate the NLPR3 inflammasome related to IL-1 and IL-6 secretion, triggering the cytokine cascade that induces severe lung damage [130].

Anakinra, a recombinant human IL-1 receptor antagonist with a short half-life (3–4 h) that neutralizes the biological activity of IL-1α and IL-1β by competitively inhibiting their binding to receptors, has been used successfully in treating MAS induced by various inflammatory disorders. IL-1α and IL-1β also increase nitric oxide production, prostaglandin, adhesion molecules, thromboxane and histamine [131], all of which contribute to CRS in the pathogenesis of COVID-19. In this respect, many clinical trials have been conducted that support the rationale for targeting augmented inflammatory processes in COVID-19 with anakinra. Outcomes are promising, though diverse use, dosages and outcomes have been observed in the various trials [132]; most studies have evaluated the use of anakinra in the early stages of COVID-19 infection, with substantial differences in their design and results [133].

A case series of 11 COVID-19 patients with evidence of acute hypoxic respiratory failure and signs indicative of increased cytokine release showed that administration of anakinra, given early in the clinical course of respiratory failure, benefited in terms of a significantly reduced risk of requiring mechanical ventilation [134]. Further data deriving from a cohort study have shown a beneficial role of IL-1 inhibition with anakinra in reducing the need for mechanical respiratory support and also improving life expectancy in severely ill individuals with radiological evidence of bilateral pneumonia [135]. Data from another cohort study that included 21 patients with severe COVID-19 infection showed that administration of anakinra led to a decrement in clinical and laboratory inflammatory signs, though without a subsequent reduction in the duration of ICU admission or mechanical respiratory support [136]. Benefit from IL-1 blockade with anakinra was not observed in another RCT in non-ventilated COVID-19 patients with hypoxic respiratory failure and high CRP [137]. It is likely that the dosage of anakinra chosen was inadequate, given that observational cohort studies with higher doses of this drug reported more promising effects [138]. However, an RCT (SAVE-MORE) published in 2021, which introduced the same therapeutic regimen of anakinra (subcutaneous, 100 mg once daily) revealed an impressive 55% improvement in 28-day mortality in patients with moderate and severe COVID-19 preselected for high concentrations of soluble urokinase plasminogen activator receptor [139]. Therefore, patient selection might be critical to detect those who benefit from anakinra treatment. In the same direction, Kharazmi et al. have shown that the administration of anakinra in hospitalized COVID-19 patients with hypoxemia resulted in a significantly reduced length hospital of stay as well as a decreased need for mechanical respiratory support [140]. Nevertheless, no significant improvement in clinical outcomes was observed when anakinra was administered to COVID-19 patients with mild-to-moderate pneumonia [141]. A recent patient-level meta-analysis substantiated the benefit from anakinra administration in COVID-19 patients with moderate-to-severe respiratory tract infection and increased inflammatory markers, specifying this benefit in cases with the IL-1 antagonist being given without parallel administration of dexamethasone [142]. Additionally, an updated systematic review and meta-analysis of six studies referring to individuals with moderate-to-severe pneumonia depicted a favorable effect of anakinra in selected COVID-19 patients, with a relative mortality risk reduction of 53% (pooled hazard ratio 0.47 (95% confidence intervals 0.34, 0.65)) [143]. The impact of methylprednisolone and low-dosage anakinra combined therapy in patients with hyper-inflammatory syndrome also revealed a statistically significant result in the treatment arm in terms of mortality rate, as the number of deaths at day 28 decreased by 13.9% compared to 35.6% in the control arm [144].

As in the case of IL-1, IL-6 amplification also induces CRS [145]. IL-6 is a key cytokine associated with severity and mortality of COVID-19-induced disease [146]. It is considered a reliable indicator for distinguishing the prognosis and severity of COVID-19-induced disease [147]; its high concentrations appear to be a good predictor of oxygen requirement, intubation and poor prognosis in affected patients [148]. IL-6 pro-inflammatory action is associated with pneumonia and a negative correlation has been found between IL-6 and partial oxygen pressure in arterial blood and peripheral oxygen saturation indicating respiratory failure and requiring mechanical ventilation [149,150]. Thus, IL-6 estimation is recommended in the treatment of COVID-19-induced disease [151].

Tocilizumab, an IL-6 receptor blocker humanized monoclonal antibody (mAb), has been approved for the management of specific autoimmune disorders and has also gained United States Food and Drug Administration (FDA) approval for the treatment of CRS resulting from some types of immunotherapy [152]. Therefore, it has been tested for the treatment of severe COVID-19-related disease [153]. In a single-center study that included 100 individuals with severe COVID-19 pneumonia, IL-6 blockade with tocilizumab resulted in significant clinical improvement, though no control group was used in the above study [154]. Another clinical study that focused on the effects of tocilizumab in critically ill patients with COVID-19 respiratory failure treated with mechanical ventilation found that tocilizumab led to a decrease in the mortality rate among these patients. Increased incidence of superinfections in patients treated with tocilizumab did not seem to affect the above favorable effect in mechanically ventilated patients due to COVID-19 pneumonia [155]. However, further findings from recent RCTs have shown that tocilizumab did not substantially prevent death or mechanical ventilation [156] and solely decreased the risk for mechanical ventilation without having favorable effects on 28-day mortality [137,157]. Nevertheless, a meta-analysis of recent three RCTs showed an incremental benefit with tocilizumab [158,159,160,161]. Another recent meta-analysis also indicates that tocilizumab, by preventing mechanical ventilation and death and increasing the discharge rate, may be a suitable therapeutic regimen for patients with COVID-19 mild/moderate or severe disease [161].

A widespread scientific discussion is also in progress with regard to the potential use of convalescent plasma (CP) in high-risk patients with SARS-CoV-2 infection. This pertains to the timely delivery of CP acquired from COVID-19-recovered donors to patients presenting with acute onset of disease symptoms. Specifically, CP, as an approach in passive antibody immunotherapy, has been intensively investigated during the COVID-19 pandemic, owing to its strong scientific basis supported by earlier successes in other pandemics, such as the 1918 influenza pandemic and outbreaks of influenza A (H1N1), avian influenza A (H5N1), Ebola and/or other viral infections [162]. Studies of the efficacy and safety of CP in treating both severe COVID-19 and earlier courses of infection have reported promising results [163,164]. Following these studies, on 23 August 2020, the FDA has announced that CP therapy can be used for critically ill COVID-19 patients as an emergency investigational new regimen [165]. A recent randomized, single-blind trial using a total of 511 patients who attended an emergency department within 7 days showed no benefit from CP in terms of delay/prevention of disease progression, nor any improvement regarding illness severity or number of hospital-free days [166]. However, there is evidence that timely (within 72 h of disease onset) administration of high-titer plasma from recovered donors to older individuals with signs of mild COVID-19 disease can offer a significant reduction in disease progression. This effect was shown to be dose-dependent and, in addition, the earlier the administration, the better the clinical outcomes that have been observed [164]. In this respect, recent systematic reviews and meta-analyses reported decreased mortality during the course of COVID-19 with CP treatment over the standard regimen when given early and at high titers, with tolerable side effects [167,168]. Likewise, two additional recent meta-analyses showed that CP significantly decreases the risk of all-cause mortality and duration of mechanical ventilation in severe or critical COVID-19-induced disease [169,170]. Considering that CP exhibits a similar safety profile as standard plasma, CP should be applied with the onset of infectious disease outbreaks, including COVID-19, SARS, MERS, Zika virus and Ebola [171]. Nevertheless, since other relative data showed no clear benefits of CP treatment in terms of mortality, the necessity for mechanical ventilation, ICU admissions and/or other measures of clinical improvement, [172,173], further research is needed to elucidate its effect on the above important clinical outcomes.

There is a debate regarding the potential utility of hyperimmune immunoglobulin and plasmapheresis as adjunctive treatment modalities. The rationale of administering CP-derived hyperimmune globulin relies on the pathophysiological ground of providing high titers of antibodies against SARS-CoV-2 in a timely manner [174]. As the SARS-CoV-19 pandemic continues its course and currently available vaccines have crucial limitations in controlling community transmission, commercial immunoglobulin products derived from healthy plasma donors become gradually enriched in anti-SARS-CoV-2 antibodies [175]. Currently, since antibody concentrations in the general population are still low [175], the plasma collected and the products produced cannot yet be reflecting hyperimmunity. Anti-SARS-CoV-2 hyperimmune immunoglobulin is classically prepared from pools of 100–1000 L from CP donors. Hyperimmune immunoglobulin products display a high titer of neutralizing antibodies against SARS-CoV-2 in a standardized and concentrated product [176]. This signifies an advantage over treatment with CP. Apart from neutralization, hyperimmune globulin exhibits additional antiviral actions, which have been reported for CP. These include antibody-dependent cellular phagocytosis [177], antibody-dependent cellular cytotoxicity [178] and complement-mediated cytotoxicity [179]. These well-described functions of antibodies might add to neutralizing actions and enable non-neutralizing antibodies or antibodies with poor-neutralizing capacity to block or clear infection. Hyperimmune globulin effectiveness is being examined in ongoing randomized clinical trials in inpatient (intravenous administration) and outpatient settings (subcutaneous administration). In this respect, relative recent data indicate that intravenous administration of hyperimmune immunoglobulin in severe and critical COVID-19 patients is safe, increases survival and reduces disease progression [180].

Plasmapheresis consists of a method of extracting plasma from an individual for therapeutic purposes and can be used in two different ways: either for removing pathological factors, e.g., autoantibodies or mediators of inflammation, from an individual’s circulation [181,182] or in order to collect antibody-rich CP from recovered COVID-19 patients with high antibody titers which can then be processed to promptly provide a severely ill patient with hyperimmune antibodies against the viral pathogen [174,183]. By introducing plasma exchange, Adeli et al. showed that clinical symptoms and CRS were controlled in eight severe COVID-19 patients with antiviral and corticosteroid therapies failure [184]. Likewise, Morath et al. conducted a study by using plasma exchange in five COVID-19 patients suffering from refractory fever associated with respiratory failure, vasopressor-dependent shock and increased IL-6. The results showed that plasma exchange decreased serum levels of CRP, IL-6, ferritin, LDH and D-dimers [185]. In another study, in six COVID-19-associated meningoencephalitis patients, plasmapheresis reduced serum ferritin and improved disease-connected clinical signs [186]. Therefore, plasmapheresis in patients with severe forms of COVID-19 can effectively improve the clinical symptoms of the disease and reduce inflammatory mediators in patients, and thus further studies are needed.

Novel research into the management of COVID-19 has also suggested the potential use of virus-neutralizing monoclonal antibodies as a means of limiting viral load and preventing the worsening of clinical signs and symptoms. Notably, monoclonal antibodies, a type of passive immunotherapy, are laboratory-created molecules that mimic or improve the body’s natural immune reactions to invaders, such as tumors or infections. Since monoclonal antibodies are engineered to directly target an important portion of the infectious process, they offer an advantage over conventional approaches to antiviral therapy. They are created by exposing white blood cells to a specific viral protein and cloning it to mass-generate antibodies against a specific virus. Monoclonal antibodies have been developed even before the COVID-19 pandemic, when they were introduced to treat various viral disorders, including Ebola and rabies [187]. As SARS-CoV-2 utilizes its spike protein to bind to ACE2 receptors in order to enter human cells, diverse neutralizing monoclonal antibodies have been formed that target the spike protein in an effort to prevent the virus from infecting human cells [188]. The FDA has granted Emergency Use Authorization for three neutralizing monoclonal antibodies for the treatment of selected non-hospitalized patients with COVID-19, namely, LY-CoV555 (bamlanivimab ± etesevimab), REGEN-COV (casirivimab + imdevimab) and sotrovimab. They are recombinant neutralizing human monoclonal antibodies targeting the spike protein of SARS-CoV-2. These neutralizing monoclonal antibodies are given with a single intravenous administration on an outpatient basis to COVID-19 patients usually in an emergency department, an infusion center or other outpatient settings (such as the patient’s home or nursing home). Currently, there have been several randomized trials estimating the effect of the early use of neutralizing monoclonal antibodies on the risk of progression to severe COVID-19 in terms of hospital admission and the risk of mortality. Specifically, LY-CoV555 has been evaluated in individuals with a recent diagnosis of mild or moderate SARS-CoV-2 infection. A significant drop in viral load was observed with one of the three evaluated doses of LY-CoV555, while an overall better clinical course and subsequently lower rate of hospitalization was observed in these patients as compared to the placebo arm [189]. Recently published data also showed that REGEN-COV reduced clinical presentation of COVID-19 infection, admissions to hospital and all-cause mortality [190]. Moreover, relative data confirm the benefits of timely administration of REGEN-COV in reducing hospitalization rates in COVID-19 patients [191]. Likewise, a recent RCT showed that subcutaneous REGEN-COV prevents symptomatic and asymptomatic COVID-19 infection in previously uninfected household contacts with infected people and decreases the duration of both symptomatic disease and high viral load [192]. Finally, current evidence suggests sotrovimab’s safety and efficacy in limiting the risk of disease progression in individuals with mild-to-moderate SARS-CoV-2 infection, as it decreases the risk of disease progression [193].

A list of additional antiviral agents has also been considered to attack COVID-19, especially during the initial phase of the infection.

Remdesivir is classified as a broad-spectrum antiviral with potential antiviral activity against a variety of RNA viruses [194]. It was the first FDA-approved antiviral against SARS-CoV-2 as well as the first FDA-approved COVID-19 treatment. Remdesivir has been the most thoroughly investigated antiviral therapeutic agent and has also been widely field-tested in real-world clinical settings. A double-blind placebo-controlled ACTT-1 trial recruited 1062 patients and randomly assigned them to placebo treatment or treatment with remdesivir. This study reported an association between remdesivir administration and both clinical improvement and a lack of progression to more invasive respiratory intervention in patients receiving noninvasive and invasive ventilation at randomization [195].

Another randomized open-label trial that included 584 individuals diagnosed with moderate COVID-19 pneumonia demonstrated that a 10-day course of remdesivir did not offer significant clinical improvement compared to standard of care, while the 5-day course was associated with evidence of improved clinical status relative to the standard-of-care group [196]. Nevertheless, in a mortality trial recommended and funded by the World Health Organization, remdesivir failed to present a benefit in terms of the overall incidence of deaths, commencement of ventilation or duration of hospitalization [197]. Nevertheless, the Grein study reported that 68% of patients hospitalized for severe COVID-19 showed clinical improvement with remdesivir [198]. The Beigel study also showed that remdesivir was superior to placebo in shortening the time to recovery and lowering the incidence of respiratory tract infection [195]. Conversely, other studies have not shown significant clinical improvement [199] or a difference in clinical status in moderate COVID-19 patients treated with remdesivir compared to regular standard of care [196]. Finally, Olender et al. detected in patients with severe COVID-19 that, by day 14, remdesivir therapy was associated with a greater degree of recovery and reduced odds of death versus standard-of-care treatment [200]. Thus, it seems that remdesivir works in certain groups of COVID-19 patients.

Molnupiravir represents another antiviral agent, initially destined for the treatment of influenza. This medication, given orally, targets the RNA-dependent RNA polymerase and consequently affects the viral replication process [201]. There are already available data regarding molnupiravir’s safety and tolerability [202] and several further clinical studies are underway [201]. Data from a phase II/III trial showed that molnupiravir is unlikely to demonstrate a clinical benefit in hospitalized patients. In outpatients, molnupiravir reduces the risk of hospitalization or death by approximately 50% compared to placebo for patients with mild or moderate COVID-19, according to an interim analysis of a phase III study [203].

In an effort to uncover potential ways to control inflammation in SARS-CoV-2 infection, the Janus kinase pathway has been under investigation for the past 18 months. Jak inhibitors, including baricitinib, imatinib and tofacitinib, have been evaluated for the above purpose. Confinement of the NF-κB signaling pathway, prostaglandin E2 stimulation, JAK–STAT signal blockade and interception of viral endocytosis have been described as the main mechanisms via which JAK inhibitors, can prove beneficial in reducing viral load and restricting excessive cytokine release [204,205].

As far as imatinib is concerned, data from a recently published RCT have shown that, despite some favorable findings in terms of duration of mechanical ventilation and survival rates, no benefit was observed regarding the primary outcome of cessation of mechanical ventilation and adjuvant oxygen for a sustained period longer than 48 h [206]. A recent meta-analysis shows that monoclonal antibodies, including imatinmib as well as baricitinib, sotrovimab and bamlanivimab, baricitinib, imatinib and sotrovimab, may offer benefits for treating severe or non-severe COVID-19 patients [59].

Tofacitinib, an oral JAK-1 and JAK-3 inhibitor, has been linked to reduced mortality risk or respiratory failure in hospitalized COVID-19 patients, 28 days after initiation of administration, as compared to placebo [207]. Low dosages of tofacitinib may also improve COVID-19 disease symptoms in home settings [208].

Baricitinib constitutes a promising JAK-1 and JAK-2 inhibitor that may provide attenuation of both viral endocytosis and uncontrollable cytokine expression and release. Baricitinib presents favorable effects in reducing the incidence of intubation and ICU admissions, decreasing the mortality rate as well as reducing the overall hospitalization period in COVID-19 patients with moderate pneumonia [209,210]. Baricitinib’s increased affinity for AAK1, a member of the numb-associated kinase group, is considered the main advantage of this agent over the other JAK inhibitors [211]. Baricitinib’s efficacy and safety has also been investigated in conjunction with remdesivir, resulting in verdicts of its superiority regarding recovery time and clinical improvement when compared to remdesivir alone, especially in individuals with increased oxygen demands [212]. That having been said, some attention should be given to the slightly raised risk of adverse events, mainly upper respiratory tract infections and herpetic infections [211,213]. Other recent data, however, suggest that adding baricitinib did not significantly decrease mortality in hospitalized patients exhibiting clinical progression of COVID-19 disease despite combined therapy with tocilizumab plus corticosteroids. Nevertheless, the combination of baricitinib plus tocilizumab was not associated with an augmented risk of secondary infections or thromboembolic incidents [214].

Interferons (IFNs) are crucial to stimulating the innate immune response against viral infections. Specifically, IFN-β has been shown to intensively inhibit the replication of coronaviruses, such as SARS-CoV-1, in cell culture, while IFN-α and -γ were found to be less effective in this setting [215]. There is an indication that patients with higher susceptibility to ARDS display deficiency in IFN-β. For example, infection with coronaviruses impairs IFN-β expression and synthesis, thereby allowing the virus to escape the innate immune response [216]. Currently, many ongoing relative clinical trials are considering the potential effects of IFN-β-1a, including in combination with remdesivir [National Institute of Allergy and Infectious Diseases (NIAID)]. Nevertheless, a recent phase III trial reported that INF-β-1a combined with remdesivir was not superior to remdesivir alone in hospitalized patients with COVID-19 pneumonia (NCT04492475). A multicenter, adaptive, randomized blinded controlled trial designed to assess the safety and efficacy of investigational therapeutics for the treatment of COVID-19 in hospitalized adults (ACTT-3) [217] and those therapies currently administered via inhalation for the treatment of patients with confirmed SARS-CoV-2 infection [218]. As further relative data become available, a more thorough understanding of whether and under what circumstances IFN-β-1a is beneficial to COVID-19 patients should be acquired.

The pediatric population has also been described to suffer from SARS-CoV-2 infection. However, a mild course of COVID-19 has been reported in the vast majority of children as compared to adults [219]. Apart from the typical presentation of viral pneumonia, there has been recorded a very rare children-specific clinical entity termed multisystem inflammatory syndrome in children (MIS-C), which consists of a state of overactivation of the inflammatory response leading to multiple organ involvement, resembling the clinical course of Kawasaki’s disease (KD) or toxic shock syndrome (TSS) [220]; pediatric disorders, including KD, TSS, bacterial sepsis and MAS, have been reported after recovering from COVID-19 and many patients display myocardial dysfunction and coronary artery involvement in addition to gastrointestinal and systemic symptoms [221,222,223].

Two COVID-19 vaccines may have possible protective effects in children and adolescents, though awareness is needed to monitor potential adverse effects after injection. The US CDC recommends each child of 12 years and older should get a COVID-19 vaccination to help protect against COVID-19 disease as a potential strategy to inhibit the pandemic. The agency also supports the co-administration of COVID-19 vaccines and other routine pediatric vaccines [224]. However, increasing cases of myocarditis and pericarditis have been recently reported in both adolescents and young adults in the post-COVID-19 vaccination period with the introduction of the Pfizer–BioNTech mRNA vaccine (BNT162b2) and the Moderna mRNA vaccine (mRNA-1273), which are connected with increased risks for myocarditis and pericarditis (Moderna: ROR = 2.91, 95% CI = 2.21–3.83; Pfizer–BioNTech: ROR = 5.37, 95% CI = 4.10–7.04) [225]. Whether children below 12 years of age ought to be vaccinated against COVID-19 currently is a matter of intense debate. The rather low risk of acute COVID-19 disease in children along with concerns raised over the impairments from vaccination and disease signify that the balance of vaccination risks and benefits in this age group is more complex. Moreover, the appearance of novel variants of concern demands constant re-evaluation of the risks and benefits [226]. Initial short estimations suggest that although the long-term outcome of acute pediatric myocarditis is relatively good, there is a risk of chronic left ventricular dysfunction (CLVF); complete regression and CVLVD and late deaths owing to cardiac failure occur in 73% and 27% of cases, respectively [227]. Since recent data indicate that younger patients hospitalized for acute myocarditis without a history of cardiac disorder are associated with a long-term increased risk of heart failure and death [228], further relative research is warranted to elucidate the long-term pediatric/adolescent acute myocarditis risk in the post-COVID-19 vaccination period.

To date, despite the extended literature with regard to the pharmacological management of COVID-19 in adults, only limited data are available for the treatment of pediatric patients when needed. Children with mild-to-moderate disease are generally treated with solely supportive measures that include hydration, clinical monitoring for signs of decompensation and supplemental oxygen therapy when indicated. More specific treatment in children is considered for more complicated forms of COVID-19. Antiviral therapy with remdesivir is considered to treat severe disease in children above 12 years of age and more than 40 kg in weight [229]. Off-label use of dexamethasone is also recommended in severe cases of COVID-19, although sufficient evidence in pediatric populations is lacking as of yet [230]. IFN-α, administered via spray or nebulization, has been described as a potential agent aiming to attenuate viral load and shorten the disease course [230]. Plasmapheresis and CP therapy represent additional therapeutic options that could be considered in pediatric COVID-19 patients treated in tertiary centers. However, the evidence regarding these therapies is drawn from studies on adults [230]. The use of monoclonal antibodies at early stages has also been under discussion in selected children hospitalized with COVID-19 [229]. The management of MIS-C still remains more challenging and immunomodulatory therapy with the IL-1 receptor antagonist anakinra or the IL-6 receptor antagonist tocilizumab (off-label use) can be considered. Referral to a specialized multidisciplinary pediatric team is recommended in such cases whatsoever [229], considering the possible need for inotropes or vasoactive agents in children presenting with signs of heart failure and hypotension [231]. Finally, hyperimmune immunoglobulin and aspirin have been proposed for the management of MIS-C [220].

## 4. Non-Pharmacological Management of the SARS-CoV-2 Epidemic

The spread of COVID-19 has led to an appropriate combination of non-pharmaceutical measures and public health system measures aiming to reduce the transmission of SARS-CoV-2, its resultant disease and death rates and health system overload [232]. At the beginning of the pandemic the lack of experience and scientific evidence about the virus, as well as the lack of pharmacological options, forced governments, in consultation with scientific advisory committees, to enforce non-pharmacological interventions (NPI). Remarkably, the adapted NPIs were associated with several adverse effects, besides any questionable benefits [232]. The most globally widespread NPIs implemented include, besides vaccination, handwashing, physical distancing, use of facemasks (indoors and outdoors) and lockdowns, which should be reviewed for their efficacy, as they may be associated with negative social, religious, economic and health consequences.

### 4.1. Handwashing

Handwashing has always been one of the most effective ways of keeping diseases at bay, reducing healthcare-associated infections and enhancing patient safety [233]. For enveloped viruses, such as SARS-CoV-2, soap molecules decompose the lipid envelope of the virus and deactivate it [234]. Numerous methods have been recommended by CDC and the Environmental Protection Agency for decontamination using standard disinfectants, such as hand sanitizers, alcohol, a diluted solution of sodium hypochlorite or household bleach [235]; daily hand hygiene with alcohol-based hand sanitizers might exhibit the lowest rates of skin barrier disruption and the highest reduction of bacteria and fungi colony-forming count [236]. Such disinfection methods appear to control local transmission, whether human-to-human, fomites or airborne transmission; air cleaners fitted with filters along with ultraviolet light and upper room fixtures of ultraviolet germicidal irradiation using short-wavelength ultraviolet C light has proven effective against SARS-CoV-2 [237].

### 4.2. Physical Distancing

At the beginning of the COVID-19 pandemic, healthcare specialists made swift accommodations to offer reliable and safe care, including emphasizing remote access to permit physical distancing [238]. The measure of social and physical distancing aims to slow the spread of disease by stopping chains of transmission of SARS-CoV-2 and preventing the propagation of the epidemic. The term “physical distancing” is being used to emphasize the need to stay at least 6 feet away from others (about two arm-lengths—1.8 m) and the importance of maintaining physical space when in public areas, especially if someone is at higher risk of severe disease [235,239,240]. However, other studies suggest that even more caution may be warranted as it has been discovered that the virus may travel not just on heavy, short-range droplets but also in much finer—and more mobile—aerosol clouds [241].

### 4.3. Facemasks

One of the strategies in response to SARS-CoV-2 has been the utilization of masks. Masks might possibly reduce SARS-CoV-2 transmission by preventing the spread of the virus from infected people to others (source control/exhalation isolation), by protecting wearers (protective effect/inhalation protection) or both. Transmission can occur before symptoms develop and this is the reason why source control is believed to be one of the primary mechanisms for reducing SARS-CoV-2 transmission [242]. For exhalation isolation, both surgical and N95 masks may be effective in reducing the spread of respiratory diseases, the latter being much more effective. For inhalation protection, air-filtering respirators, such as N95 masks, can filtrate contaminants, bacteria and other matter before they reach the nose and mouth and are more efficient when it comes to virus penetration inhibition than surgical masks [235].

The utilization of masks in response to SARS-CoV-2 is of importance, at least for indoor environments due to the greater risk of airborne transmission, especially when ventilation is poor [235]. Improving ventilation by means of increased air exchange with outdoor air and air filtration constitute important components in addition to the aforementioned established practice [243]. Outdoors mask-wearing remains controversial, as it might be a discouragement for outdoor activities and worsen social isolation [243]. CDC suggests that in general there is no need to wear a mask outdoors except when involved in crowded events [240]. A randomized controlled trial, DANMASK-19, concluded that wearing surgical masks to supplement other public health measures did not reduce the SARS-CoV-2 infection rate among participants by more than 50% in a community with modest infection rates, some degree of social distancing and uncommon general mask use [242,244,245]. Moreover, noticeable side effects should also be taken into consideration. Wearing a mask may give a false sense of security and reduce compliance with other more effective control measures [246,247]. For example, the volume of speech between people wearing masks is compromised and they unconsciously come closer. Wearing a mask makes the exhaled air go into the eyes and this generates an impulse to touch the eyes, particularly in the pediatric population [247]. Facemasks make breathing more difficult, increase inspiratory and expiratory flow resistance [248] and decrease alveolar ventilation [249]. A fraction of carbon dioxide previously exhaled is inhaled again, therefore breathing frequency and deepness increase [247]. Masks also give rise to a humid habitat where the virus can remain active and accumulate, causing an increase in viral load and therefore defeating innate immunity [247,250].

The disadvantages listed above should be weighed against the ability of the mask to reduce SARS-CoV-2 transmission, especially in indoor environments. Wang et al. came to the conclusion that correctly wearing masks of all kinds, despite their different designs, functions and effectiveness, will reduce, to a large degree, the overall risks of COVID-19 and enhance general protection from coronavirus [235]. Long-time use of a facemask might cause pressure sores on the ears and nose bridge if the mask does not fit the face shape of the mask wearer [251]. Quality control of the material and design of facemasks should not be overlooked. Similar to other articles of dress, various sizes and designs of facemask to promote comfort for the wearer might help to increase facemask-wearing in the present COVID-19 pandemic situation.

### 4.4. Lockdowns

Lockdowns were defendable choices in the beginning of the pandemic due to the rapid spread of the disease and overwhelmed health systems in relation to the limited knowledge about the virus’ morbidity and mortality. Lockdowns of all kinds are, comparatively, the most draconian non-pharmaceutical interventions. When implemented successfully, they are thought to decrease disease transmission by limiting human contact at scale [252]. On the other hand, it needs to be acknowledged that SARS-CoV-2 is being transmitted primarily in domestic environments, in which case lockdowns may actually facilitate transmission by prolonging human interaction at home. Historical archival analysis of 43 cities in the 1918–19 influenza pandemic has shown a strong association between lockdowns and delay of the temporal effects of a pandemic, reduced overall and peak attack rates and peak mortality rates, as well as reduced cumulative deaths [253]. Earlier implementation and longer lockdowns were also associated with reduced total mortality [253]. Due to the potential adverse effects associated with highly restrictive non-pharmacological measures, it is increasingly recognized that their postulated benefits deserve careful consideration. Global lockdown has had an acute effect on quality of life in most people, particularly in urban areas [254]. During the past year, when lockdowns were extensively introduced and enforced, many adverse effects have been documented, including hunger, increases in opioid-related overdoses, increases in non-COVID-19-related diseases [255,256], domestic abuse [257], mental health and suicidality [258,259,260] along with a host of economic consequences with dire health implications [261,262].

Further research is needed to estimate the extent of such lockdown-related critical adverse effects in relation to important human life-supporting practices, including social reassurance processes, active participation in holy services as well as positive psychological influences on human life, the interruption of which triggered identified negative economic and psychological effects (i.e., the aforementioned domestic ferocity, depression, desperateness and augmented suicide) among global populations. Interruption of the societal, religious, working and economic processes owing to COVID-19 had undesirable impacts on social and psychological well-being, and attending the management of such processes appears to be a critical element in controlling such disastrous pandemics [30].

### 4.5. “Safepass”/Vaccine Passport/Vaccine Certificate

Following more than one year of restricted life, imposing high economic and social burdens, the European Commission launched arbitrarily a vaccine passport to facilitate safe free movement within the EU for those who are vaccinated, recovered or tested [263]. Governments worldwide invented vaccine passports suggesting more freedom of domestic movements and international travel [264]. The proof of immunization to severe acute respiratory syndome coronavirus 2 (SARS-CoV-2) is used as a supplement to efforts aiming to safely reopen society [264]. Vaccine passports, although questionable, will eventually allow only a certain rate of people to travel, attend large gatherings, access public venues and return to work without compromising personal safety and public health [265]. Despite the aforementioned presumed advantages, there has been a great debate on the disadvantages and the ethical considerations surrounding vaccine certificates. There are many concerns about the constitutionality of a “safepass”, as it is indirectly leading to a violation of integrity. Equity concerns exist due to the exclusion of those who remain unvaccinated, who cannot get the vaccine for various reasons including medical history and who do not have access to it [263,265,266,267,268]. As vaccine passports are digital and require access to private medical records, there are important questions around internet access, the costs of acquiring and maintaining passports as well as data privacy and protection [263,265,266]. The extent of protection conferred by vaccination, particularly against new variants, is not yet well understood, nor is the potential for viral transmission by people who have been vaccinated [266,268]. “Safepass” implementation also has economic consequences and does not help businesses to recover because the limited activity does not cover operating costs [263]. Future research should inform policy makers of the potential benefits and adverse effects of this newly developed control measure.

## 5. Conclusions

The COVID-19 pandemic has changed our lives in an unprecedented way. A series of measures for the effective and successful confrontation of this extraordinary challenge is undoubtedly required. Acquired immunity is eventually the best way to prevent disease transmission and propagation of the epidemic. Convalescents are characterized by a robust and long-lasting immunity, which is presumably better than the immunity offered by current vaccines. The wide variety of pharmacological and non-pharmacological interventions provide some optimism for the future, albeit more evidence is needed, and, up to now, they have not been able to control the pandemic. Therefore, the freedom of each individual to be able to choose the best option for his/her preventive or therapeutic scheme should be maintained. Politicians, policy makers and particularly physicians should always be guided by the moral obligation to implement medical and public health practice based on the safe option that is believed to confer a benefit that outweighs the possible risks, i.e., “*ofeleein i mi vlaptein*”.

## Data Availability

Not applicable.
